# Preparation of Anti-Zearalenone IgY and Development of an Indirect Competitive ELISA Method for the Measurement of Zearalenone in Post-Fermented Tea

**DOI:** 10.3390/foods12244478

**Published:** 2023-12-14

**Authors:** Taotao Qiu, Huayi Zhang, Hongtao Lei, Lin Zhang, Yaqiong Zhang, Xing Shen, Biyun Xu, Jialin Zhu, Wentao Xiao, Jixu Zheng, Jiahong Chen

**Affiliations:** 1 College of Physical Education and Health, Guangxi Normal University, Guilin 541004, China; gxqiutaotao@mailbox.gxnu.edu.cn (T.Q.); wahyeez@163.com (H.Z.); z2066511489@163.com (L.Z.); xubiyun1700@163.com (B.X.); jialin-zhu_07@stu.gxnu.edu.cn (J.Z.); x15885924472@163.com (W.X.); 15963063853@163.com (J.Z.); 2 Guangdong Provincial Key Laboratory of Food Quality and Safety, National-Local Joint Engineering Research Center for Machining and Safety of Livestock and Poultry Products, College of Food Science, South China Agricultural University, Guangzhou 510642, China; hongtao@scau.edu.cn (H.L.); heyi_2017@163.com (Y.Z.); shenxing325@163.com (X.S.)

**Keywords:** zearalenone, post-fermented tea, egg yolk immunoglobulins, indirect competitive enzyme-linked immunosorbent assay

## Abstract

Post-fermented tea (PFT) is one of the most commonly consumed beverages worldwide. Rapid microbial growth and significant changes in the microbial composition of PFT during processing and storage pose a potential risk of contamination with mycotoxins such as zearalenone (ZEN). Screening for ZEN contamination in a simple, rapid, and inexpensive manner is required to ensure that PFT is safe for consumption. To monitor ZEN in PFT, ZEN was conjugated with bovine serum albumin to prepare egg yolk immunoglobulins (IgY). A specific indirect competitive enzyme-linked immunosorbent assay (ic-ELISA) based on IgY was developed and validated. ZEN was extracted with acetonitrile and water (50:50, *v*/*v*) containing 5% acetic acid and purified using a mixture of primary and secondary amines and graphitized carbon black to remove matrix interference from the PFT samples. Under optimal conditions, the linear range of this assay was 13.8−508.9 ng mL^−1^, the limit of detection was 9.3 ng mL^−1^, and the half-maximal inhibitory concentration was 83.8 ng mL^−1^. Cross-reactivity was negligible, and the assay was specific for ZEN-related molecules. The recovery rate of ZEN in the control blanks of PFT samples spiked with a defined concentration of ZEN of 89.5% to 98.0%. The recovery and accuracy of the method were qualified for PFT matrices. No significant differences were evident between the results of the actual PFT samples analyzed by high-performance liquid chromatography and ic-ELISA. The collective data indicate that the developed ic-ELISA can be used for the rapid and simple detection of ZEN in PFT products.

## 1. Introduction

Post-fermented tea (PFT), also known as dark tea, is commonly consumed in Southeast Asia, China, and other countries [1]. PFT is uniquely produced by microbial fermentation that involves a long aging period [2]. The product has unique biological activities and health benefits, including the prevention of cardiovascular disease and antioxidant, anti-obesity, antibacterial, and antidiabetic activities [3]. Because some PFT raw materials are usually used as coarse old tea leaves of the tea plant, as well as for improper tea garden management, fresh leaf picking, tea processing, circulation, and storage, unknown fungi and indigenous microbial species are commonly present or naturally inoculated during PFT processing and storage, which might result in mycotoxin contamination [4,5]. Over the last decade, many mycotoxins have been identified in Pu-erh, Liupao, and Fuzhuan teas. They include aflatoxins (AFs), ochratoxin A (OTA), and zearalenone (ZEN) [6]. In another study, deoxynivalenol, enniatins, AFs, sterigmatocystins, OTA, and ZEN were detected in 20 Pu-erh tea samples obtained from different European countries [7]. A survey of the mycotoxin contamination of Pu-erh tea marketed in Latvia reported 35% ZEN contamination in Pu-erh tea samples [8]. Among these mycotoxins, ZEN is a very common and deleterious toxicant in PFT, which is consistent with the results of our previous long-term monitoring of mycotoxins produced in naturally contaminated PFT. ZEN is a low-molecular-weight resorcylic acid lactone toxin produced by *Fusarium culmorum* and *F. graminearum* [9]. ZEN has numerous toxic effects on humans and animals, including estrogenic syndrome, genotoxicity, and carcinogenicity [10,11]. Monitoring the ZEN contamination of PFT is important for minimizing potential health risks.

Various analytical methods have been developed to accurately detect ZEN in food and foodstuffs. Methods suitable for both laboratory and commercial detection have been discussed in previous reports and can be categorized into three main types: thin-layer chromatography, chromatography, and immunoassay methods [12]. Chromatographic methods include liquid phase and mass spectrometry methods. Liquid chromatography–mass spectrometry is most often used to detect ZEN [13]. Layer chromatography has also been employed to detect ZEN [14]. However, this method is outdated and is less commonly used. Concurrently, some ZEN immunoassays have been developed [15,16]. According to the literature, after eliminating interference from the sample matrix, ZEN in edible and medicinal coix seeds can be analyzed using an indirect competitive enzyme-linked immunosorbent assay (ic-ELISA) [17]. High-throughput immunoassays are highly sensitive and rapid. However, the use of traditional mammal-derived antibodies is limited by their low production, false positive reactions, and animal welfare concerns [18,19]. These shortcomings necessitate the discovery of new antibodies from multiple species to develop novel methods to reduce false positives, increase sensitivity and ease of preparation, and simplify analysis.

Egg yolk immunoglobulins (IgY), also known as egg yolk antibodies, are widely present in poultry and amphibians. IgY consists of two heavy chains and two light chains with a molecular mass of approximately 180 kDa. The molecular mass of the heavy chain is 62–67 kDa and that of the light chain is 22–25 kDa. The IgY heavy chain consists of a variable region and four constant regions. The light chain consists of a variable region and a constant region. The Fc fragment in the antibody is the main hydrophobic group [20]. IgY has specific binding antigenic elements such as serum immunoglobulin G [21]. IgY from chicken egg yolk is an inexpensive and convenient source of polyclonal antibodies [22]. Blood collection and damage to the source material/organism are not required, which conforms to the principles of animal welfare and has the advantages of simple preparation, low cost, easy storage, and large-scale production [23]. Additionally, IgY does not activate the complement system and does not interact with human Fc receptors or mammalian rheumatoid factors, thus avoiding non-specific reactivity [24]. Consequently, IgY is a valuable and stable tool for detecting various macromolecules, bacteria, and proteins [25]. However, owing to the lack of a corresponding T-cell epitope, it is difficult for small molecules to directly stimulate the animal immune system to produce specific antibodies compared to macromolecules [26]. The key to preparing IgY using small-molecule mycotoxins is the design of active artificial antigens.

In this study, ZEN was selected as the hapten and conjugated with bovine serum albumin (BSA) to stimulate the formation of anti-ZEN IgY. An anti-ZEN IgY antibody was used to develop an ic-ELISA specific for ZEN in PFT. After evaluating the sensitivity and specificity of the obtained IgY, ic-ELISA was used for ZEN determination in PFT samples. In addition, the results were compared with those obtained using high-performance liquid chromatography (HPLC) to validate their reliability and accuracy.

## 2. Materials and Methods

### 2.1. Reagents and Materials

Pu-erh, Liupao, and Fuzhuan teas were purchased from a market in Guangxi, China. The zearalenone, zearalanone, α-zearalenol, β-zearalenol, AFB_1_, and OTA standards were purchased from J and K Scientific Ltd. (Beijing, China). Freund’s complete adjuvant (FCA), Freund’s incomplete adjuvant (FIA), ovalbumin (OVA), BSA, O-(Carboxymethyl) hydroxylamine hemihydrochloride (CMO), N, N-dimethylformamide (DMF), N-hydroxylsuccinamide (NHS), 1-ethyl-3-(3-dimethylaminopropy) carbodiimide hydrochloride (EDC), and peroxidase-labeled goat anti-chicken IgY (IgY-HRP) were purchased from Sigma-Aldrich (St. Louis, MO, USA). Pyridine, tween-20, and polyethylene glycol 6000 (PEG 6000) were obtained from Meryer Chemical Technology Co., Ltd. (Shanghai, China). Ethyl acetate, acetonitrile, acetic acid, acetone, n-hexane, ethanol, hydrochloric acid, ammonium sulfate, and sodium sulfate (Na_2_SO_4_) were purchased from Sinopharm Chemical Reagent Co., Ltd. (Shenyang, China). Phosphate-buffered saline (PBS) powder was purchased from Beyotime (Shanghai, China). Magnesium sulfate (MgSO_4_), primary and secondary amines (PSA), octadecylsilane, and graphitized carbon black (GCB) were purchased from Macklin Ltd. (Shanghai, China). All other chemicals and organic solvents were of analytical grade.

### 2.2. Synthesis of ZEN Conjugates

ZEN (5.0 mg) was dissolved in pyridine (1.5 mL) containing CMO (10 mg) and stirred for 2 h at room temperature. Aliquots of the reaction solution (20 μL) were separated by TLC using n-hexane/ethyl acetate/acetic acid (5:5:1, *v*/*v*) as an expansion agent to isolate the reaction product. The ZEN conjugates were extracted with ethyl acetate, and Na_2_SO_4_ was added and stirred for 8 h at room temperature in the dark. The ethyl acetate extract was evaporated until dry by using a rotary evaporator. The resulting mixture was then dissolved in acetone (1 mL). The acetone solution was separated by TLC using an expansion agent (n-hexane/ethyl acetate/acetic acid, 50:50:1, *v*/*v*) to obtain ZEN-CMO. ^1^H-NMR and ^13^C-NMR were subsequently employed to identify ZEN-CMO.

### 2.3. Preparation of Immunogen

Artificial ZEN antigens were prepared using the active ester method. ZEN-CMO (12 mg), 11.2 mg EDC, and 9.3 mg of NHS were sequentially dissolved in 900 μL DMF at room temperature overnight. Then, 12.2 mg BSA was added dropwise to the activated hapten solution, and the mixture was stirred for 8 h at room temperature. The reaction solution was dialyzed for 3 d to remove small molecules and obtain ZEN-CMO-BSA as an immunogen. ZEN-CMO-OVA was prepared using an active ester method for antigen coating. UV spectroscopy was used to determine the conjugation efficiency of ZEN-CMO with carrier molecules.

### 2.4. Generation of IgY

We previously reported that AFB_1_-GA-BSA can be used as an immunogen to generate anti-AFB_1_ IgY. Anti-ZEN IgY was prepared in the present study following this previous protocol [27]. ZEN-CMO-BSA (400 μg) and an equal volume of FCA were used to immunize chickens. After the fifth immunization, eggs were collected from each chicken. Anti-ZEN IgY was purified using the ammonium sulfate precipitation method [28]. After washing, the eggs were soaked in a 0.05% bromogeramine solution for 20 min. The egg whites were removed using a yolk separator. The yolks were collected in a beaker. Eight times the volume of acidic distilled water was added to the beaker, and stirring was performed using a magnetic mixer. The pH of the solution in the range of 5.0−5.2 was adjusted with 1M HCl, and the final volume ratio of egg yolk to water was 1:9. Then, the acidic egg yolk solution was placed in the refrigerator at 4 °C overnight. The acidic egg yolk solution was centrifuged at 12,000 rmp for 20 min, and the supernatant was filtered with a 0.22 µm membrane. Water-soluble yolk extract was added to a saturated ammonium sulfate solution and incubated at room temperature for 30 min. The solution was centrifuged at 12,000 rpm for 10 min at room temperature. The precipitate was dissolved in 18% PEG6000 solution to obtain anti-ZEN IgY and stored at −20 °C. The purity of the anti-ZEN IgY antibody was determined using sodium dodecyl sulfate–polyacrylamide gel electrophoresis. The concentration of the separation glue was 12% and that of the concentrated glue was 5%.

### 2.5. ic-ELISA

ic-ELISA was performed using a previously described ELISA protocol [29]. Briefly, the ELISA plates were coated with ZEN-CMO-OVA (100 µL/well). After incubating at 37 °C overnight, the plates were washed twice with PBST and blocked with blocking buffer. Anti-ZEN IgY and ZEN (0.5, 5, 10, 50, 150, 500, 1000, and 2000 ng mL^−1^) were then added to the wells. After incubation for 40 min, the plates were washed five times with PBST. IgY-HRP was added to each well. TMB solution was added, and the mixture was incubated for 10 min. After termination of the reaction, the absorbance of the reaction solution was determined at 450 nm using a microplate reader (Thermo Fisher Scientific, Waltham, MA, USA). The inhibition rate (I) was expressed as I= (A_0_ − A)/A_0_ × 100, where A and A_0_ are the absorbances of the ZEN standard and negative wells, respectively. Data were analyzed using a four-parameter logistic function plotted using Origin 8.5 (Origin Lab Corp., Northampton, MA, USA). ZEN(IC_10_) was defined as the limit of detection (LOD), IC_20_–IC_80_ as the dynamic detection range, and IC_50_ as the performance.

### 2.6. Specificity Assessment of Anti-ZEN IgY

The specificity of the anti-ZEN IgY was assessed based on the IC_50_ value and cross-reactivity. ZEN, zearalanone, α-zearalenol, β-zearalenol, AFB_1_, and OTA were chosen for evaluation under the optimized ic-ELISA conditions. Cross-reactivity was calculated (%) as (IC_50 (ZEN)_/IC_50 (compound)_) × 100.

### 2.7. PFT Sample Pre-Treatment Optimization

Liupao teas were selected as the representative PFT samples for ZEN isolation. ZEN was extracted and purified using a previously described method with some modifications [30,31]. Ten grams of the ground PFT sample was placed into a triangular flask, followed by the addition of 100 mL of acetonitrile and water (50:50, *v*/*v*) containing 5% acetic acid. After 2 h of shaking, the supernatant was decanted and centrifuged. Two-milliliter aliquots of the supernatant were dispensed into 5 mL centrifuge tubes containing 250 mg of magnesium sulfate. Two absorbents, PSA and GCB, were used to optimize purification. After centrifugation of the extract for 5 min at 10,000 rmp, the supernatant was filtered through a 0.22 μm microfilter membrane for HPLC analysis and immunoassay.

### 2.8. Validation of ic-ELISA

To reduce the matrix effect, the following method was used to prepare the matrix solutions of blank PFT samples. The matrix solution was diluted 5-, 10-, and 20-fold in a working buffer. ZEN (50 ng mL^−1^) was prepared at three different dilutions for the ELISA. A suitable dilution was determined by comparing the recovery rates. If the ELISA results for ZEN were similar to the true concentration after the matrix solution was diluted to a certain magnification, this dilution or a higher dilution was used to eliminate the matrix effect.

Recovery rates were used to evaluate the accuracy of the ELISA. The recovery rates of the developed ELISA were evaluated by spiking fixed amounts of ZEN (20, 100, and 200 ng mL^−1^) into ZEN-free PFT. Analyses were conducted in triplicates for each concentration. Recovery rates were determined by comparing the ZEN concentrations of the spiked samples with those of the standard solutions.

Furthermore, to ensure the accuracy and precision of the ic-ELISA, each pretreated sample (Liupao, Fuzhuan, and Pu-erh teas) solution was subjected to ZEN analysis using ic-ELISA and HPLC. ZEN was quantified by HLPC, as previously reported, with some modifications [32]. After sample pre-treatment, ZEN levels in the PFT samples were determined using an LC-20A prominence HPLC system (Shimadzu, Kyoto, Japan) with a fluorescence detector at an excitation wavelength of 274 nm and an emission wavelength of 440 nm. The mobile phase consisted of methanol/acetonitrile/water in a ratio of 8:46:46, and it was filtered through a C_18_ XBridge column (4.6 mm × 150 mm) and a 0.45 μm filter. The flow rate was set at 0.8 mL min^−1^, and the entire run time for the analysis was 20 min.

## 3. Results and Discussion

### 3.1. Identification of Artificial Antigen

ZEN has the molecular formula C_18_H_22_O_5_. The chemical structure of ZEN is a 6- (10-hydroxy-6-oxo-trans-1-undecenyl)-β-resorcylic acid lactone [33]. ZEN is a non-immunogenic hapten because of its low molecular weight, with a relative molecular weight of 318.86 [34]. A highly sensitive and specific antibody against small molecules can be prepared using hapten–protein conjugates [35]. To enhance the prepared antibodies’ susceptibility to antigens for resorcylic acid lactones, 6′-carboxymethyloxime derivatives of ZEN can be linked to carrier proteins using the active ester method [36]. Thus, CMO was chosen as the linking site to leave the resorcylic acid lactones far from the carrier protein, which increased the reliability and stability of ZEN conjugated with BSA and OVA [12]. NMR spectroscopy was used to identify ZEN-CMO haptens. In the ^1^H NMR spectrum, the chemical shift of 4.64 (2H, s) was due to the hydrogen atoms of the methylene group (-CH_2_) on the hapten spacer arm (Figure S1A). The ^13^C NMR spectrum showed signals for all 20 carbon atoms in the hapten (Figure S1B). These results indicated that the synthesis of the ZEN-CMO hapten was completed.

**Figure 1:**
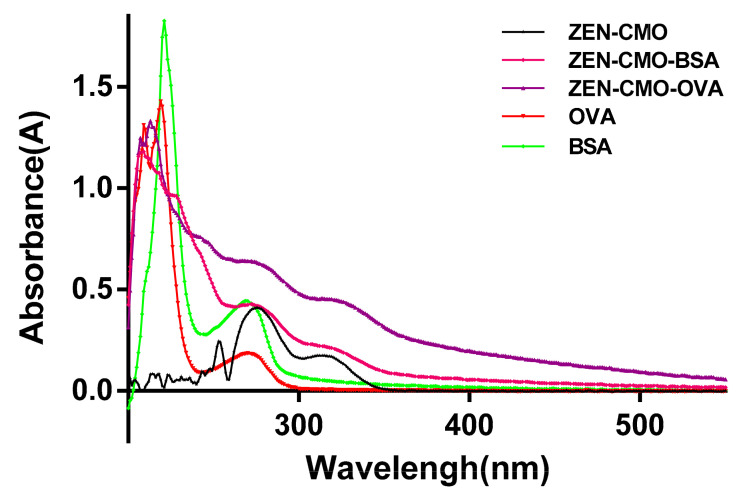
UV spectra of ZEN-CMO, ZEN-CMO-BSA immunogen, ZEN-CMO-OVA coating antigens, OVA, and BSA carrier proteins.

To increase the immune response, keyhole limpet hemocyanin, BSA, OVA, and horse serum albumin are often used as carrier proteins. BSA is the most stable and soluble albumin, with approximately 30–35 major amino groups available for conjugation with linkers, making it a popular carrier protein for weakly antigenic compounds. Thus, BSA was conjugated to ZEN-CMO as a carrier protein to prepare the immunogen. OVA, an egg albumin, was used to prepare the coating antigen and verify that the antibody was specifically targeted. These conjugations were confirmed by UV spectrophotometry. The UV spectra of the ZEN-CMO, ZEN-CMO-BSA immunogen, ZEN-CMO-OVA coating antigens, BSA, and OVA carrier proteins are shown in Figure 1. The maximum absorbance peak of ZEN-CMO was observed at 325 nm, with both OVA and BSA displaying characteristic absorbance peaks at 280 nm. After dialysis, characteristic absorbance peaks of ZEN-CMO-BSA and ZEN-CMO-OVA were observed at 280 and 325 nm, respectively, which differed from the characteristic absorbance wavelengths of the carrier proteins. These data indicated the successful synthesis of immunogens and coating antigens [37] and that the obtained ZEN-CMO-BSA and ZEN-CMO-OVA could be used for subsequent immunoassays.

### 3.2. Purification and Characterization of Anti-ZEN IgY

**Figure 2:**
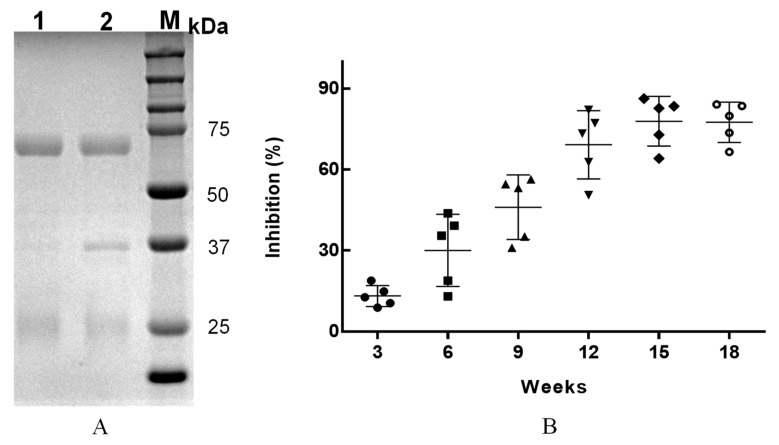
SDS-PAGE analysis of purified IgY (**A**) and inhibitory activity of anti-ZEN IgY from egg yolks of five chickens at different immunization times (**B**). The results are expressed as mean ± standard deviation of five replicates.

The purification of IgY is often limited to yolk lipids and lipoproteins. Therefore, the initial extraction step for IgY should remove as many lipids and lipoproteins as possible from egg yolks. Acidified water dilution is the most commonly used de-lipidation technique [38]. After delipidation, chromatography, precipitation, and filtration methods can be applied for further purification of IgY [25]. Precipitation methods, including ammonium sulfate and PEG precipitation, are commonly used extraction methods for IgY [39]. This approach is a simple, inexpensive, and high recovery process that efficiently removes fat [40,41]. In the present study, the yolk was diluted and extracted using acidic water. The extract was purified using 35% saturated ammonium sulfate and PEG6000. The purity of the IgY was determined via reductive electrophoresis. The disulfide bond of the antibody protein was opened under reducing conditions and divided into two bands. Three bands were clearly observed on SDS-PAGE after purification with 35% saturated ammonium sulfate (Figure 2(A2)). After further purification with PEG6000, two bands were observed by SDS-PAGE (Figure 2(A1)), corresponding to the 65 kDa heavy chain and 25 kDa light chain, as previously reported [42]. SDS-PAGE demonstrated IgY production following immunization of laying hens with ZEN-CMO-BSA. ImageJ software (version 1.53a) analysis of the gels revealed that the purity of anti-ZEN IgY was approximately 93%.

Eggs harvested after the initial immunization were used to prepare IgY and collected continuously for 18 weeks. After 18 weeks, the results of the experiment showed that egg production decreased significantly. The inhibitory activity of the anti-ZEN IgY from egg yolks was monitored during immunization. Anti-ZEN IgY was produced in the third week after the initial immunization of the laying hens. The inhibitory activity of anti-ZEN IgY markedly increased with increasing immunization time. When immunization was performed for 12 weeks, the anti-ZEN IgY produced showed a high rate of inhibition. The highest inhibition rate was observed at 15 weeks (Figure 2B). These results are consistent with those of previous studies [43]. Therefore, the anti-ZEN IgY obtained after 12 weeks was used to develop the ic-ELISA in this study.

### 3.3. Development of ic-ELISA for Detection of ZEN

**Figure 3:**
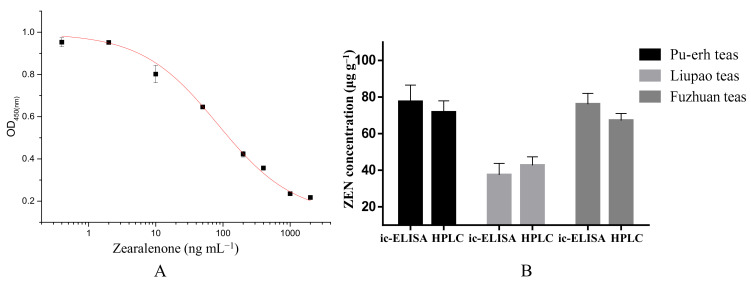
Development of the ic-ELISA for ZEN (**A**) and analysis of ZEN in real PFT samples via HPLC and ic-ELISA (**B**). The results are expressed as mean ± standard deviation of triplicate determinations. *p* > 0.05 indicates no significant difference from the detection result when compared with the ic-ELISA and HPLC group (one-way ANOVA).

The experimental parameters of ic-ELISA are the key to accurate detection. The multiple parameters included antibody dilution, coating concentration, buffer used for the competition reaction, competition time, IgY-HRP dilution, and chromogenic reaction time. The optimum dilutions for the plate-coating antigen ZEN-CMO-OVA and anti-ZEN IgY were determined using the checkerboard titration method. The optimal concentration of ZEN-CMO-OVA was determined to be 0.03 ng mL^−1^. Based on the antibody inhibitory activity results used to determine the ratio of positive hole value to negative control, the antibody (anti-ZEN IgY) dilution time was 1:2000 (*v*/*v*). The working buffer (PBS) concentration was 10 mmL^−1^, the competition time was 40 min, the exposure time for IgY-HRP was 30 min, and the chromogenic reaction time was 12 min (Table 1). The optimal performance of the method was determined by comparing the IC_50_ and dose–response curves obtained from four-parameter sigmoidal fitting [44]. Figure 3 shows the results of the ic-ELISA calibration curves constructed under optimum conditions. As shown in Table 1, the linear range for the developed ic-ELISA capable of detecting ZEN was 13.8 to 508.9 ng mL^−1^, the ZEN LOD was 9.3 ng mL^−1^, and IC_50_ of anti-ZEN IgY was 83.8 ng mL^−1^. The LOD and IC_50_ values of IgY were lower than those previously reported for fumonisin B by Tran et al. [45] and similar to those described for citrinin by Duan et al. [46]. The results were considered acceptable, as the values were below the maximum allowed amount of ZEN in cereals and cereal products set by the European Union regulations [47].

### 3.4. Determination of Specificity

Mycotoxin contamination is common in PFT [48]. Several mycotoxins have structures similar to ZEN, and other mycotoxins that contaminate PFT include AFB_1_ and OTA [49,50]. The specificity of IgY was assessed by checking cross-reactivity. Furthermore, the cross-reactivity of ZEN-related molecules and other mycotoxins that contaminate PFT was evaluated using ic-ELISA. Table 2 summarizes the IC_50_ values and cross-reactivities of anti-ZEN IgY against ZEN-related molecules and other mycotoxins. Reflecting their similar structures, zearalanone displayed an affinity for anti-ZEN IgY comparable to that of ZEN. The cross-reactivity rate with zearalanone was slightly lower (85.0%). On the other hand, the cross-reactivity was <5% for α-zearalenol and β-zearalenol and <2% for AFB_1_ and OTA compared with ZEN (Table 2). The trend of cross-reactivity in these mycotoxins was consistent with that of other ZEN polyclonal antibodies prepared using ZEN-BSA-immunized BALB/c mice [26]. The cross-reactivity results indicated that the anti-ZEN IgY against ZEN and ZEN-related molecules was specific and sensitive.

### 3.5. Validation of ic-ELISA

#### 3.5.1. Effect of Matrix

The accuracy of immunoassays may significantly interfere with matrix effects. Therefore, the elimination of matrix effects is vital. PFT naturally contains pigments, polyphenols, catechins, L-theanine, proteins, lipids, and other active ingredients [51]. These constituents affect the accuracy of the mycotoxin determinations [52]. To reduce matrix interference in the ZEN ELISA, purification was performed as previously described with some modifications [53,54]. PSA and GCB were used for purification. PSA is a common adsorbent that can adsorb fatty acids, organic acids, polar pigments, and sugars via weak anion exchange (aqueous solutions), polar interactions (nonpolar organic solvents), and complexation. GCB is graphitized carbon black consisting of six carbon atoms in a planar hexagonal shape that readily adsorbs pigments, sterols, and non-polar interferents. Although 500 mg PSA was used to minimize matrix interference, the extract appeared slightly yellow (Figure S2A). The PFT extract was colorless when co-treated with 50 mg GCB (Figure S2B). These results are consistent with prior results [55]. However, considering the potential for mycotoxin adsorption by adsorbing materials, further optimization of the purification processes for ic-ELISA is necessary.

Dilution is an important step in the development of an ELISA method required to minimize matrix effects. To reduce the matrix effect for the detection of ZEN in PFT samples and decrease the use of adsorbents, the initial extract from PFT was undiluted or diluted 5-, 10-, and 20-fold, and the recoveries were compared. The recoveries were 127.1, 115.0, 106.3, and 85.2% (Table 3). A 10-fold dilution of the initial PFT extract reduced the matrix effects to acceptable levels. Therefore, PSA, GCB, and 10-fold dilution were effective in minimizing matrix interference.

#### 3.5.2. Recovery

The applicability of the developed ic-ELISA for the detection of ZEN in PFT was tested using a recovery test. The recovery results of ZEN from PFT samples were compared with the recovery results from ZEN-spiked control blanks to assess the accuracy of the ic-ELISA method. The recovery rates of 20, 100, and 200 ng mL^−1^ ZEN ranged from 89.5 to 98.0%. The relative standard deviations (RSD) ranged from 9.9 to 12.3% (Table 3). These results indicate the high sensitivity of anti-ZEN IgY for ZEN, consistent with the results obtained using an anti-ZEN monoclonal antibody [56]. The recovery of ZEN by the developed ic-ELISA method satisfied the analysis requirements of Commission Regulation (EC) No. 401/2006 (70–120% recovery, RSD ≤ 40%) [57]. A clear matrix effect was observed in the PFT extraction solution during the optimized purification process.

#### 3.5.3. Detection of ZEN in PFT Samples

PFT is a basic beverage that is consumed daily by many people. Various PFTs include Liupao, Fuzhuan, and Pu-erh teas [58]. The three PFTs contaminated with ZEN were analyzed using HPLC and ic-ELISA. The reliability of the developed ic-ELISA was also evaluated via a comparative analysis of HPLC and ic-ELISA detection results. The retention time of the ZEN standard substances obtained by HPLC was 7.3 min. The results of the HPLC method showed good linearity for the target ZEN with a correlation coefficient of 0.9998 and a linear equation of y = 363.33x + 686.12 (Figure S3). The mean ZEN values of Pu-erh, Liupao, and Fuzhuan tea samples detected by ic-ELISA were 77.4 ± 9.1, 37.5 ± 6.4, and 76.1 ± 5.9 μg kg^–1^, respectively. The mean ZEN values detected by HPLC in the same respective order were 71.7 ± 6.2, 42.7 ± 4.7, and 67.2 ± 3.8 μg kg^–1^, respectively. No statistically significant differences in ZEN concentrations in the three types of PFTs were evident using either HPLC or ELISA (*p* > 0.05; Figure 3B). These findings demonstrate the reliability and accuracy of the developed ic-ELISA for ZEN detection in PFT products.

## 4. Conclusions

In this study, a rapid and sensitive ic-ELISA method based on anti-ZEN IgY was developed to detect ZEN in PFT. The ZEN-CMO hapten was synthesized to link carrier proteins. The anti-ZEN IgY antibody raised from chicken eggs immunized with ZEN-CMO-BSA was specific to ZEN. The performance of the developed method was evaluated by analyzing the LOD, IC_50_, linear workable range, and recovery. These results demonstrated that IgY is a favorable antibody for the detection of ZEN in PFT. The analysis of real samples confirmed a good correlation between the developed ic-ELISA and HPLC. Thus, ic-ELISA may be valuable for the rapid screening of ZEN in PFT products. These results support the implementation of local initiatives to prevent mycotoxin contamination in the beverage industry.

## Figures and Tables

**Table 1:** The optimization parameters for ci-ELISA.

Parameters	ic-ELISA
Coating antigen	ZEN-CMO-OVA
Coating concentration (ng mL^−1^) and antibody dilution	0.03/1:2000
Buffer for competition reaction	PBS (10 mM, pH 7.4)
Competition time (min)	40
HRP-rabbit anti-IgY incubation time (min)	30
HRP-rabbit anti-IgY dilution	1:5000
Chromogenic reaction time (min)	12
IC_50_ (ng mL^−1^)	83.8
Linear workable range (IC_20_–IC_80_; ng mL^−1^)	13.8–508.9
The limit of detection (ng mL^−1^)	9.3

**Table 2:** The cross-reactivity of the anti-ZEN IgY to ZEN-related molecules and other mycotoxin occurrences in PFT.

Compound	IC_50_ (ng mL^−1^)	^a^ CR(%)
Zearalenone	83.76	100
Zearalanone	>100	<85
α-zearalenol	>500	<20
β-zearalenol	>500	<20
Aflatoxin B_1_	>5000	<2
Ochratoxin A	>5000	<2

^a^ CR% was calculated using units of moles per liter for IC_50._

**Table 3:** Matrix effects and recovery of ic-ELISA performance in PFT (n = 3).

Matrix	Spiked (ng mL^−1^)	Dilution Factor	Recovery (%)	RSD (%)
Post-fermented tea	50	0	127.1	11.6
		5	115.0	7.6
		10	106.3	8.4
		20	85.2	10.4
	20	10	96.4	11.8
	100		98.0	9.9
	200		89.5	12.3

## Data Availability

The data presented in this study are available on request from the corresponding author. The data are not publicly available due to restrictions of privacy.

## Supplementary Materials

The following supporting information can be downloaded at: https://www.mdpi.com/article/10.3390/foods12244478/s1, Figure S1, The ^1^H-NMR spectrum of ZEN-CMO hapten (A) and ^13^C-NMR spectrum of ZEN-CMO hapten (B). Figure S2, Post-fermented tea extraction solution (A), extraction solution color after absorbance using only PSA (B), and extraction solution color after absorbance using PSA and GCB (C). Figure S3, HPLC chromatograms of ZEN (A) and standard curve (B).
