# Supplementary material for: Preparation of Anti-Zearalenone IgY and Development of an Indirect Competitive ELISA Method for the Measurement of Zearalenone in Post-Fermented Tea
Source: Foods. 2023 Dec 14;12(24):4478. doi: 10.3390/foods12244478 (PMC10742412; doi:10.3390/foods12244478)
Supplement: Supplementary file 1 [file foods-12-04478-s001.zip › foods-2733789-supplementary.pdf]

Supplementary materials for

Preparation of Anti-Zearalenone IgY and Development of an Indirect Competitive ELISA Method for Measurement of Zearalenone in Post-Fermented Tea

Taotao Qiu[1], Huayi Zhang[1], Hongtao Lei[2], Lin Zhang[1], Yaqiong Zhang[2], Xing Shen[2], Biyun Xu[1], Jialin Zhu[1], Wentao Xiao[1], Jixu Zheng[1], and Jiahong Chen*[,2]

[1] College of Physical Education and Health, Guangxi Normal University, Guilin, 541004, China
[2] Guangdong Provincial Key Laboratory of Food Quality and Safety/National-Local Joint Engineering Research Center for Machining and Safety of Livestock and Poultry Products, College of Food Science, South China Agricultural University, Guangzhou 510642, China
* Correspondence: authors: jiahongchen@scau.edu.cn (Jiahong Chen), Tel.: +8620 8528 3925;

This material includes the results:

The ^{1}H-NMR spectrum of ZEN-CMO hapten and ^{13}C-NMR spectrum of ZEN-CMO hapten (**Figure S1**). Post-fermented tea extraction solution, extraction solution color after absorbance using only PSA, and extraction solution color after absorbance using PSA and GCB (**Figure S2**). HPLC chromatograms of ZEN and standard curve (**Figure S3**).

3. Results and Discussion

3.1. Identification of artificial antigen

ZEN has the molecular formula $C_{18}H_{22}O_5$. The chemical structure of ZEN is a 6- (10-hydroxy-6-oxo-trans-1-undecenyl)-β-resorcylic acid lactone [33]. ZEN is a non-immunogenic hapten because of its low molecular weight, with a relative molecular weight of 318.86 [34]. A highly sensitive and specific antibody against small molecules can be prepared using hapten-protein conjugates [35]. To enhance antibodies prepared susceptible to antigens for resorcylic acid lactones, 6'-carboxymethyloxime derivatives of ZEN can be linked to carrier proteins using the active ester method [36]. Thus, CMO was chosen as the linking site to leave the resorcylic acid lactones far from the carrier protein, which increased the reliability and stability of ZEN conjugated with BSA and OVA [12]. NMR spectroscopy was used to identify ZEN-CMO haptens. In the ^{1}H NMR spectrum, the chemical shift of 4.64 (2H, s) was due to the hydrogen atoms of the methylene group ($-CH_2$) on the hapten spacer arm (Figure S1A). The ^{13}C NMR spectrum showed signals for all 20 carbon atoms in the hapten (Figure S1B). These results indicated that the synthesis of the ZEN-CMO hapten was completed.

3.5. Validation of ic-ELISA

3.5.1. Effect of matrix

The accuracy of immunoassays may significantly interfere with matrix effects. Therefore, the elimination of matrix effects is vital. PFT naturally contain pigments, polyphenols, catechins, L-theanine, proteins, lipids, and other active ingredients [51]. These constituents affect the accuracy of the mycotoxin determinations [52]. To reduce matrix interference in the ZEN ELISA, purification was performed as previously described with some modifications [53, 54]. PSA and GCB were used for purification. PSA is a common adsorbent that can adsorb fatty acids, organic acids, polar pigments, and sugars via weak anion exchange (aqueous solutions), polar interactions (nonpolar organic solvents), and complexation. GCB is graphitized carbon black consisting of six carbon atoms in a planar hexagonal shape that readily adsorbs pigments, sterols, and non-polar interferents. Although 500 mg PSA was used to minimize matrix interference, the extract appeared slightly yellow (Figure S2A). The PFT extract was colorless when treated with 50 mg GCB (Figure S2B). These results are consistent with prior results [55]. However, considering the potential for mycotoxin adsorption by adsorbing materials, further optimization of the purification processes for ic-ELISA is necessary.

Dilution is an important step in the development of an ELISA method required to minimize matrix effects. To reduce the matrix effect for the detection of ZEN in PFT samples and decrease the use of adsorbents, the initial extract from PFT was undiluted or diluted 5-, 10-, and 20-fold, and the recoveries were compared. The recoveries were 127.1, 115.0, 106.3, and 85.2% (Table 3). A 10-fold dilution of the initial PFT extract reduced the matrix effects to acceptable levels. Therefore, PSA, GCB, and 10-fold dilution were effective in minimizing matrix interference.

3.5.3. Detection of ZEN in PFT samples

PFT is a basic beverage that is consumed daily by many people. Various PFTs include Liupao, Fuzhuan, and Pu-erh teas [58]. The three PFTs contaminated with ZEN were analyzed using HPLC and ic-ELISA. The reliability of the developed ic-ELISA was also evaluated by comparative analysis of HPLC and ic-ELISA detection results. The retention time of the ZEN standard substances obtained by HPLC was 7.3 min. The results of the HPLC method showed good linearity for the target ZEN with a correlation coefficient of 0.9998 and a linear equation of $y = 363.33x + 686.12$ (Figure S3). The mean ZEN value of Pu-erh, Liupao, and Fuzhuan tea samples detected by ic-ELISA was 77.4 ± 9.1, 37.5 ± 6.4, and 76.1 ± 5.9 μg kg^{-1}, respectively. The mean ZEN values detected by HPLC in the same respective order was 71.7 ± 6.2, 42.7 ± 4.7, and 67.2 ± 3.8. No statistically significant differences in ZEN concentrations in the three types of PFTs were evident using either HPLC or ELISA ($p > 0.05$; Figure 3B). These findings demonstrate the reliability and accuracy of the developed ic-ELISA for ZEN detection in PFT products.

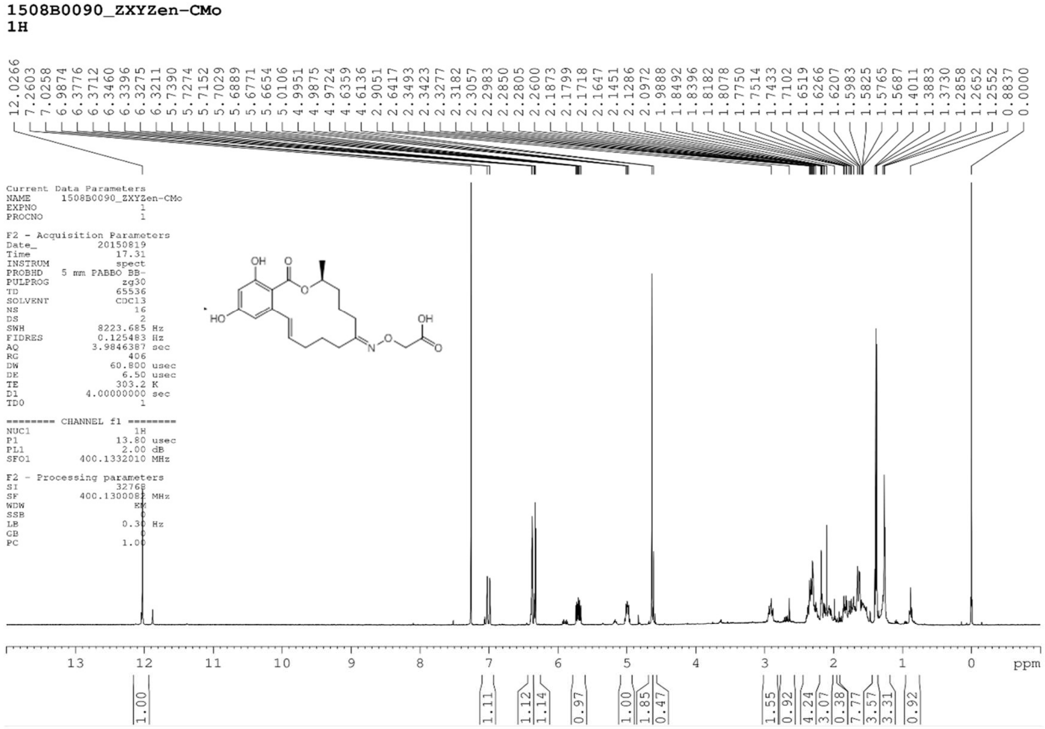

A

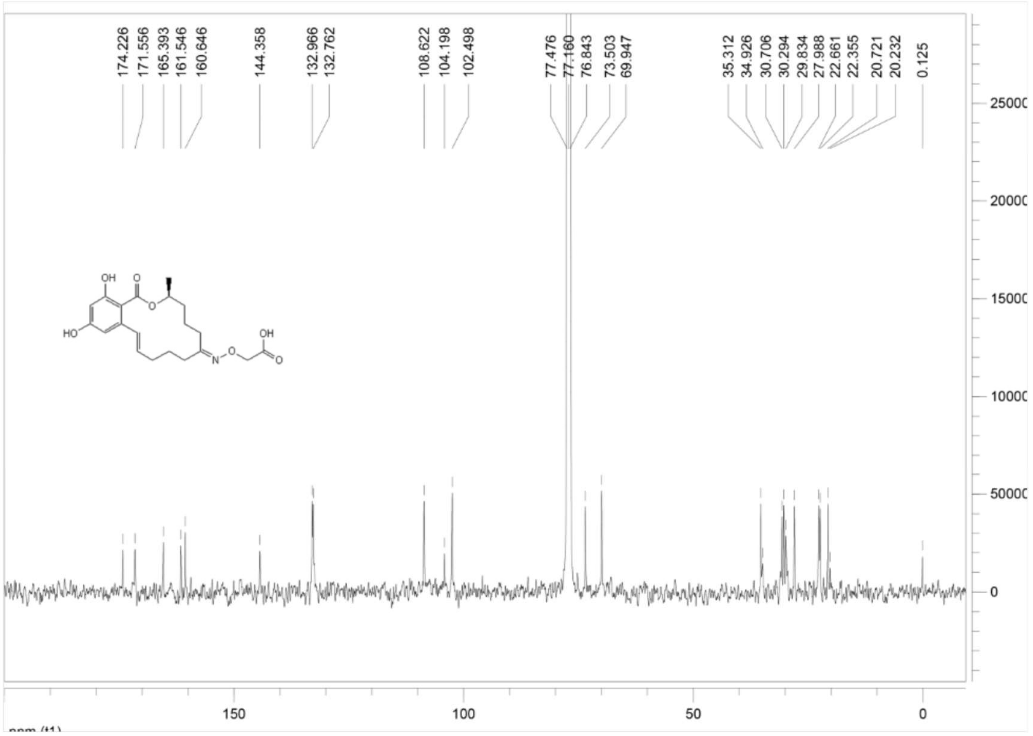

B

Figure S1. The ^{1}H-NMR spectrum of ZEN-CMO hapten (A) and ^{13}C-NMR spectrum of ZEN-CMO hapten (B)

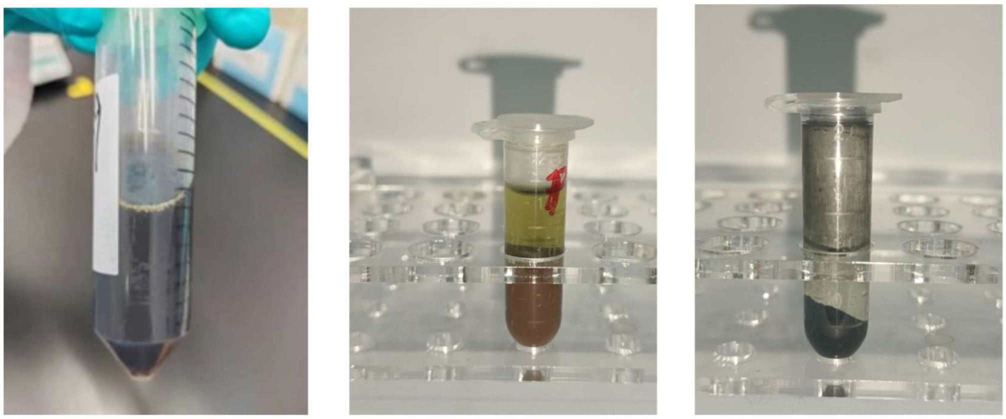

A B C

Figure S2. Post-fermented tea extraction solution (A), extraction solution color after absorbance using only PSA (B) , and extraction solution color after absorbance using PSA and GCB (C)

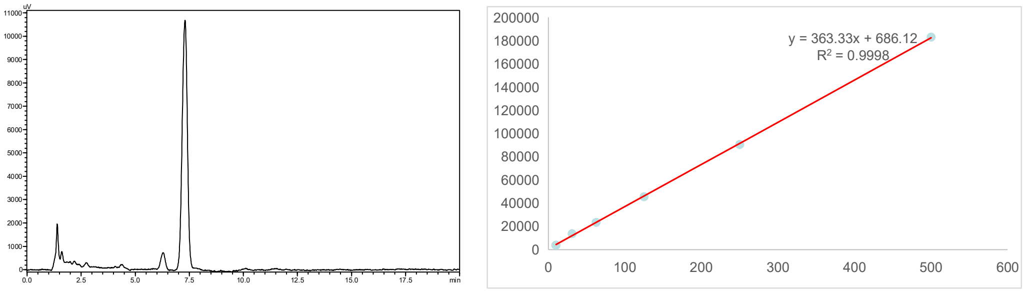

A B

Figure S3. HPLC chromatograms of ZEN (A) and standard curve (B)
